# Physical Exercise Combined with Whole-Body Cryotherapy in Evaluating the Level of Lipid Peroxidation Products and Other Oxidant Stress Indicators in Kayakers

**DOI:** 10.1155/2014/402631

**Published:** 2014-04-17

**Authors:** Paweł Sutkowy, Beata Augustyńska, Alina Woźniak, Andrzej Rakowski

**Affiliations:** ^1^The Chair of Medical Biology of Ludwik Rydygier Collegium Medicum in Bydgoszcz, Nicolaus Copernicus University in Toruń, Karłowicza 24, 85 092 Bydgoszcz, Poland; ^2^The Chair and Department of Biochemistry of Ludwik Rydygier Collegium Medicum in Bydgoszcz, Nicolaus Copernicus University in Toruń, Karłowicza 24, 85 092 Bydgoszcz, Poland; ^3^“Zawisza” Civilian and Military Sports Union, Gdańska 163, 85 915 Bydgoszcz, Poland

## Abstract

The influence of exercise combined with whole-body cryotherapy (WBC) on the oxidant/antioxidant balance in healthy men was assessed. The study included 16 kayakers of the Polish National Team, aged 22.7 ± 2.6, subjected to WBC (−120°C–−145°C; 3 min) twice a day for the first 10 days of a 19-day physical training cycle: pre exercise morning stimulation and post exercise afternoon recovery. Blood samples were taken on Day 0 (baseline) and on Days 5, 11 and 19. The serum concentration of malondialdehyde (MDA), conjugated dienes (CD), thiobarbituric acid reactive substances (TBARS), protein carbonyls, vitamin E, urea, cortisol, and testosterone were determined, along with the glutathione peroxidase (GPx) activity, the total antioxidant capacity (TAC), and morphological blood parameters. On 5th day of exercise/WBC, the baseline GPx activity decreased by 15.1% (*P* < 0.05), while on 19th day, it increased by 19.7% (*P* < 0.05) versus Day 5. On Day 19 TBARS concentration decreased versus baseline and Day 5 (by 15.9% and 17.4%, resp.; *P* < 0.01). On 19 Day urea concentration also decreased versus 11 Day; however, on 5th and 11th days the level was higher versus baseline. Combining exercise during longer training cycles with WBC may be advantageous.

## 1. Introduction

Cryotherapy involves applying extremely low temperatures (between −100°C and −160°C) to the body surface for 1 to 3 minutes. Such low temperatures are applied uniformly to the entire body surface (whole-body cryotherapy, WBC) or locally and are generated using the vapour of liquid nitrogen or liquid synthetic air [1]. Cryotherapy has been used for many years in sports to treat injuries and prevent overtraining [2, 3], as well as in the treatment of many diseases due to its analgetic, antioedematous, and anti-inflammatory effect [4]. A rapid increase in body temperature after leaving the cryogenic chamber and intensive cutaneous blood flow (tissue overperfusion lasting for several hours) induce the removal of metabolites and inflammation mediators from damaged tissues. WBC also positively affects the central nervous system (CNS) by decreasing anxiety and stress while increasing the CNS resistance to exhaustion, which may be related to the increased level of beta-endorphins [1]. However, WBC may also induce oxidative stress [1, 5]. One of the sources of ROS during WBC is a reaction catalyzed by xanthine oxidase resulting in the initial ischaemia occurring during body exposure to extremely low temperatures, as well as hyperaemia occurring after leaving the cryogenic chamber [6]. During WBC, ROS may also be generated through the oxidation of catecholamines: even a single cryogenic chamber session increases the levels of adrenalin and noradrenalin in the blood serum of young men and women [1]. The higher ROS generation during WBC sessions may also be a result of stimulation of the metabolism of brown adipose tissue which has a high content of mitochondria and cytochromes [1]. Exercise is another factor that stimulates oxygen metabolism and leads to an increased level of oxygen-derived free radicals [5]. The results of postexercise oxidative stress are, for example, microdamage of skeletal muscles and connective tissues (e.g., joint cartilages, ligaments) [7], as well as increased peroxidation of lipids forming muscle fibres and also as a result of post-WBC ROS activity, blood plasma lipids (LDL fraction), and erythrocyte membranes [5, 8]. Despite the disadvantageous effects of oxidative stress, a controlled increase in ROS concentration may cause adaptive changes involving improvements in the antioxidant capacity of the organism [9]. Many authors postulate positive adaptive changes as a result of using WBC during physical exercise and indicate WBC as a recovery or stimulation method that is being increasingly employed by athletes [1, 5, 9]. Thus, the aim of this study was to determine the effect of a total of 20 WBC sessions combined with physical exercise on the oxidant/antioxidant balance in high-level kayakers from the Polish National Team. In blood serum of sportsmen, the concentration of lipid peroxidation products (MDA, CD, and TBARS), protein carbonyls, vitamin E, urea, cortisol, and testosterone, as well as the activity of GPx and the TAC, were determined. Additionally, selected morphological blood parameters were measured: the count of red blood cells (RBC) and white blood cells (WBC) which were divided into neutrophils and lymphocytes, as well as the concentration of hemoglobin (HGB).

## 2. Material and Methods

### 2.1. Study Subject

The study involved 16 kayakers of the Polish National Team (age 22.7 ± 2.6 years, body height 184.3 ± 5.2 cm, and body weight 86.0 ± 4.9 kg). The athletes prepared for the World Championships in Duisburg (held on 22–31 August, 2013) between 22 July and 9 August, 2013 (19 days) in the Olympic Preparation Centre in Wałcz, Poland. During that period, the kayakers were subjected to strict control regarding their diet, exercise, and recovery by the team of trainers, that is, coaches, a physician, and two sport dieticians. The athletes performed specialized physical training two times a day—in the morning and afternoon. The intensity range of the exercise bouts was characterized by measuring lactate acid concentration using portable analyzer. The schedule of the training is presented in Table 1. The study was approved by the Bioethics Committee of Ludwik Rydygier Collegium Medicum in Bydgoszcz, Nicolaus Copernicus University in Torun, Poland (number KB 370/2013). The athletes provided their written consent for the participation in the study.

### 2.2. Experimental Design

During the first 10 days of the training cycle, the kayakers combined their strict exercise schedule with two WBC sessions per day. The first session was conducted in the morning, after breakfast, immediately before the first exercise bout (preexercise morning stimulation), while the second session was conducted in the afternoon, following a short rest and supper after the second (last) exercise bout (postexercise afternoon recovery). The temperature in the cryogenic chamber was gradually decreased every day from −120 to −145°C. Before every entry into the chamber, the participants remained in an adaptive vestibule for 30 seconds at −60°C. Every WBC session was 3 minutes long (excluding the stay in the adaptive vestibule). Blood samples for analysis were taken from the basilic vein at 4 time points: on Day 0 (baseline—the day before the start of the training camp), Day 5 (twice a day WBC for 5 days), Day 11 (first day without WBC), and Day 19 (no WBC for 9 successive days of the training camp). Blood samples were collected every time at midday, between the first exercise bout and the dinner.

### 2.3. Determination of the Concentrations of MDA, CD, TBARS, and Vitamin E

The levels of MDA and vitamin E were determined using high-performance liquid chromatography (HPLC), while the TBARS level was determined using the spectrophotometric method by Buege and Aust [10] as modified by Esterbauer and Cheeseman [11]. The method was also used for the preparation of serum samples for MDA quantification. The CD level was determined using the spectrophotometric method described by Sergent et al. [12]. The analytical performance of the methods used for MDA and vitamin E assessment was satisfactory with the intra-assay coefficient of variation (CV) between 5.6% and 10.4% and the interassay CV between 4.6% and 13.2%. As regrads the CD and TBARS determination methods, the ranges of intra- and interassay CV were 7.5% to 11.2% and 3.6% to 12.2%, respectively.

Vitamin E quantification was conducted by mixing 20 *μ*L working solution of internal standard (tocopheryl acetate, 186 *μ*g/mL) with 200 *μ*L serum. Protein denaturation was induced by shaking the investigated solution with 800 *μ*L acetonitrile. Once centrifuged, the supernatant was filtered using an SPE system (Captiva 2 *μ*m) into glass tubes and 4 mL hexane was added to perform extraction. Subsequently, the samples were shaken, centrifuged, and frozen at −80°C for approximately 45 min. The frozen hexane fraction containing vitamins was decanted into clean tubes and evaporated to dryness under nitrogen at 40°C. Then the sample was dissolved by adding 100 *μ*L phase, mixed ultrasonically, and finally injected into an HPLC system using a syringe. The detection was conducted using a UV-Vis detector at the wavelength *λ* = 292 nm. The concentration of vitamin E was expressed as *μ*g/L of serum.

Determination of the TBARS concentration was conducted by mixing 0.5 mL serum with 4.5 mL reaction mix consisting of 0.375% thiobarbituric acid (TBA) and 15% trichloroacetic acid (TCA) in 0.25 N HCl. The samples were incubated on a water bath for 20 min at 100°C to optimize the conditions for the MDA-TBA reaction. Subsequently, the samples were cooled down and centrifuged at 4°C for 15 min at 2000 ×g. After centrifugation, supernatant was collected. The detection was conducted at the wavelength *λ* = 532 nm. TBARS consist mainly of MDA; therefore, for the sake of simplicity, the TBARS level in serum was expressed as the MDA level (nmol/mL).

Properly prepared serum samples for MDA quantification were separated on an HPLC system using a C18 (250 mm) column. The detection was conducted using a UV-Vis detector. The concentration of the investigated compound was determined using the WorkStation Polaris software. The MDA level in blood serum was expressed as nmol/mL.CD are generated in the process of lipid peroxidation as a result of double bond regrouping after a hydrogen atom is removed from a residue of a polyunsaturated fatty acid. They form a characteristic absorbance peak at the wavelength *λ* = 233 nm. The CD level was expressed as absorbance units per mL of serum (10^−1^ Abs./mL).

### 2.4. Determination of TAC, Protein Carbonyls Concentration, and GPx Activity

The intra-assay and interassay control imprecision, as CV% obtained for the methods used in the determination of TAC and protein carbonyls, was 6.5–10.2% and 8.6–11.9%, respectively. For the GPx determination method, the interassay CV was between 6.7% and 9.1%, and the intra-assay CV was between 3.3% and 10.3%. TAC and protein carbonyls were determined using commercial ELISA kits by Cell Biolabs, Inc. The TAC test involved the reduction of Cu^2+^ ions to Cu^+^ ions by the antioxidants present in the sample. The quantity of antioxidants in the sample was directly proportional to the concentration of the newly formed Cu^+^ ions that reacted with a chromogen, forming coloured products. The absorbance of the solution was then measured at the wavelength *λ* = 490 nm and compared with the absorbance values on the calibration curve, which enabled the estimation of the antioxidant levels in the investigated sample. The calibration curve was generated based on the same procedure but using known concentrations of uric acid as an antioxidant. TAC in the serum sample was expressed as the concentration of copper-reducing equivalents (*μ*M CRE).

The quantification of protein carbonyls in the sample was based on their binding on a 96-well plate in 2-hour incubation at 37°C, followed by their detection using appropriate antibodies and estimation of their quantity from a standard curve based on the oxidized and reduced bovine serum albumin (BSA) standards. The concentration of protein carbonyls in blood serum was expressed as *μ*mol/mg.

The activity of GPx was determined using the method described by Paglia and Valentine [13]. The method is based on the decomposition of hydrogen peroxide by GPx at 20°C with the concurrent oxidation of reduced glutathione. Oxidized glutathione is then reduced in a reaction catalyzed by glutathione reductase. Reduced nicotinamide adenine dinucleotide phosphate (NADPH) is a coenzyme in this reaction and turns into an oxidized form, which causes a change in light absorbance at the wavelength *λ* = 340 nm. The GPx activity was expressed as U/L of serum.

### 2.5. Morphological Blood Parameters, Urea, and Hormones

An additional analysis of blood cell count (RBC, WBC) and HGB concentration, as well as the levels of urea, cortisol, and testosterone, was conducted. The morphological parameters were determined using the Sysmex XS 800i hematology analyzer. The concentrations of testosterone and cortisol were measured via the competition method using commercial kits by Roche. In turn, the urea concentration was assayed via the kinetic method with urease using commercial reagents by Roche as well. The assays had the intrarun CV ranging from 5.7% to 7.3% and the interrun CV ranging from 4.6% to 8.3%.

### 2.6. Statistical Analysis

The obtained results were tested for the normality of their distribution (the Kolmogorov-Smirnov test) and the homogeneity of variance (Levene's test). The main statistical analysis was represented by the ANOVA test with post hoc analysis (Tukey's range test). Moreover, Pearson's product-moment correlation coefficients between the investigated parameters were evaluated. The results were expressed as the mean ± standard deviation and differences at a significance level *P* < 0.05 were considered as statistically significant.

## 3. Results

The study demonstrated a statistically significant decrease in the baseline GPx activity by 15.1% as compared with the activity measured on 5th day of the training cycle involving physical exercise and WBC (*P* < 0.05), while after Day 19, it increased by 19.7% (*P* < 0.05) versus Day 5. Moreover, on 19th day, 9 days after the discontinuation of cryotherapy, a statistically significant decrease in the TBARS level was found as compared with the baseline value and the level detected on 5th day of exercise combined with WBC (by 15.9% and 17.4%, resp.; *P* < 0.01). On 19th day, significantly lower urea level versus 11th day was also found (*P* < 0.05), whereas on 5th and 11th days, the values were higher than baseline (*P* < 0.05 and *P* < 0.01, resp.; Table 2).

Throughout the experiment, no statistically significant changes in the levels of MDA, CD, protein carbonyls, vitamin E, cortisol, and testosterone were found in the blood serum of the study subjects. No TAC changes in the blood serum of the athletes (Table 2) and no changes in the hematological parameters were observed either (Table 3) (*P* > 0.05).

Moreover, the study indicated many statistically significant linear correlations through the whole experiment: at the baseline time point of the study, between TAC and GPx (*r* = −0.697, *P* < 0.05); on Day 5 between vitamin E and MDA (*r* = −0.645, *P* < 0.05), TBARS and vitamin E (*r* = −0.608, *P* < 0.05), and between TBARS and TAC (*r* = −0.710, *P* < 0.01); on Day 19 between MDA and TAC (*r* = −0.634, *P* < 0.05), as well as between TBARS and TAC (*r* = −0.683, *P* < 0.01).

## 4. Discussion

The obtained results show a possible profitable effect of exercise combined with WBC. Combining exercise and WBC may potentially improve sports performance because of the prolongation of exercise duration or intensity. Probably, it may result from maintenance of oxidant/antioxidant balance.

In the study, on the 5th day of physical exercise combined with WBC sessions, a decrease in the GPx activity by 15.1% versus baseline was observed (*P* < 0.05). The lower activity of GPx is a manifestation of the decreased activity of antioxidant mechanisms in the studied sportsmen. In turn, on 19th day of the training cycle (the 9th day of the exercise bouts without WBC), the GPx activity increased by 19.7% versus 5th day (*P* < 0.05), but concurrently it did not change in a statistically significant manner relative to baseline (Table 2). It demonstrates a certain degree of disturbance in oxidant/antioxidant balance during the first ten days of the training cycle associated with WBC and its recovery at the end of the cycle, that is, during nine successive days of exercise bouts following the discontinuation of WBC sessions. Both the physical exercise undertaken by the athletes and the whole-body effect of extremely low temperatures may be the source of the increased generation of ROS. During aerobic exercise, the main source of ROS is the respiratory chain, whose natural by-products are free radicals [7]. Physical exercise intensifies the metabolism of oxygen. Endurance exercise increases the demand of oxygen in the organism between 10 and 20 times. At the same time, oxygen consumption in skeletal muscles increases 100–200-fold [7]. During anaerobic exercise, that is, above the lactate threshold, the main source of ROS is xanthine oxidase produced from xanthine dehydrogenase in vascular endothelium under ischemic conditions [14]. A similar occurrence may also be observed during WBC sessions, along with the subsequent hyperemia [6]. The physical exercise undertaken by the kayakers during the training cycle was at a variable level of intensity—from aerobic to anaerobic (Table 1). The TBARS level on the 9th day of the post-WBC training cycle decreased in a statistically significant manner compared to the values measured either before the training cycle or on Day 5 of the exercise/WBC combination. Therefore, the lower level of lipid peroxidation products demonstrates the decreased level of oxidative tissue damage in kayakers through the action of these two stressors. However, cryotherapy may improve the efficiency of the TBARS elimination mechanism, whose main component is MDA, which in turn is metabolized in the liver and probably also in the skeletal muscles of physically well-trained people [5]. In turn, the results of changes in serum urea concentration in kayakers indicate that WBC also intensifies metabolism of proteins. The obtained results of GPx activity and TBARS concentration suggest that adding the effect of extremely low temperatures to physical exercise helps to maintain the balance in oxidoreduction processes. It may be explained by adaptive changes in the organism, which are described by hormesis theory. A stressor can have a tempering effect if it is used regularly for longer period at optimal intensity [15]. Such effect of WBC is also indicated by other authors, who all in all highlight the antioxidant effect of whole-body cryotherapy. In a study involving multiple WBC sessions but no physical exercise (10 WBC sessions, −130°C/3 min, once a day), in both men (*n* = 24) and women (*n* = 22), a statistically significant increase in the total antioxidant status (TAS) and the plasma level of uric acid as compared with the control group not subjected to WBC (men: *n* = 22, women: *n* = 26) was observed [16]. The results demonstrating the antioxidant properties of WBC have also been presented by other authors. Woźniak et al. [1], for example, conducted a study involving a group of professional kayakers (*n* = 20) who were subjected to a 10-day physical activity with WBC sessions conducted three times a day (1 WBC session before the first exercise bout and 2 WBC sessions before the second exercise bout, with temperatures decreasing from −120 to −140°C, 3 min) and a similar 10-day control physical activity without WBC. The study showed that the GPx activity after the 10th day of the physical activity including WBC was lower than that observed after the 10th day of the physical activity without WBC [1]. The activity of the enzyme after Day 10 of the latter physical training cycle was higher in a statistically significant manner than before this physical activity, whereas after the 10th day of the exercise bouts including three WBC sessions a day, the activity showed no statistically significant difference from that measured before the study. The article by Woźniak et al. [1] also indicates lower levels of plasma TBARS/CD and erythrocyte CD with a lower activity of the erythrocytic superoxide dismutase (SOD) after the 6th day of exercise bouts including WBC, as compared with the values obtained on the same day of the physical activity without the cryogenic chamber stimulation sessions. The authors claim that the oxidative stress induced by extremely low temperatures causes changes in the cells of the organism which may protect them against the disruption of the oxidant/antioxidant balance during physical exercise [1]. Other data indicate an anti-inflammatory effect of cryotherapy (5 sessions/week, once a day, −110°C/2 min) [17]. The authors demonstrated a statistically significant decrease in the levels of proinflammatory cytokines and an increase in the levels of anti-inflammatory cytokines/chemokines in the blood of the Italian national rugby team members, where *n* = 10 [17]. The same authors also claim not to have observed any changes in the values of selected immune system parameters: antibodies (IgA, IgM, IgG), C-reactive protein, prostaglandin E2 (PGE2), and muscle enzymes: creatine kinase (CK) and lactate dehydrogenase (LAD) [17].

The effect of a single WBC session on the oxidation and reduction processes in the human organism has also been described. Mila-Kierzenkowska et al. [18] designed an experiment in which professional volleyball players (*n* = 18) were subjected to a single WBC session (−130°C, 2 min) immediately followed by a 40-min submaximal physical exercise on a cycloergometer and then a control exercise bout excluding WBC, conducted 2 weeks later. The authors demonstrated the antioxidant and anti-inflammatory effect of WBC: higher catalase (CAT) and SOD activity was observed after the control exercise bout than after the exercise bout preceded by a WBC session. The levels of proinflammatory cytokines, interleukin 6, and 1**β** were also higher after the control exercise bout [18]. A single WBC session (−130°C, 3 min) with no exercise involved was also administered to healthy nonathletes (*n* = 10, 21.0 ± 0.9) in whom an increase in the activity of GPx and erythrocytic glutathione reductase (R-GSSG) was observed, along with an increase in the levels of nonenzymatic plasma antioxidants (glutathione, uric acid, albumins, and extra-erythrocyte hemoglobin) [19]. The authors indicated WBC as a source of ROS but also considered cryotherapy as a factor stimulating the antioxidant defense mechanisms of the organism [19]. The only nonenzymatic antioxidant that level was determined in this study was vitamin E. No statistically significant changes in its level were observed. However, it was also demonstrated that on Day 5 of the training cycle, combined with WBC, the levels of vitamin E and TBARS correlated in a statistically significant manner (*r* = −0.608, *P* < 0.05). This demonstrates the role of vitamin E in the removal of ROS generated by physical exercise and whole-body cryotherapy. Moreover, statistically significant correlations that were found are evidence for correct physiological functions in kayakers during whole experiment: baseline—TAC versus GPx (*r* = −0.697), Day 5—vitamin E versus MDA (*r* = −0.645) and TBARS versus TAC (*r* = −0.710), and Day 19—MDA versus TAC (*r* = −0.634) and TBARS versus TAC (*r* = −0.683).

Despite the ambiguous effect of WBC on the oxidant/antioxidant balance, which depends on the conditions of the study and the characteristics of the investigated group, the authors of most papers conclude that the exposure to extremely low temperatures increases the antioxidant capacity of the organism, although it is a source of ROS at the same time. The authors unanimously emphasize that WBC does not generate any significant oxidant/antioxidant imbalance and, in the long term, according to hormesis theory, may induce adaptive changes. Therefore, the WBC sessions are beneficial for health and improve the speed of postexercise recovery [1, 15, 16, 19]. It can be supposed that the results of this study confirm this hypothesis because on 5th day a clear normalization of the observed changes was noticed—the oxidant/antioxidant balance in kayakers was recovering despite the continuance of both intensive exercise bouts and, until 10th day, WBC sessions.

## 5. Conclusions

Combining exercise with whole-body cryotherapy sessions may have a positive effect on the oxidant/antioxidant balance during physical effort.

Possible profitable effect of combining exercise with cryotherapy could extend exercise duration or intensity, thus improving sports performance.

## Figures and Tables

**Table 1:** The weekly course of the training cycle combining exercise and cryotherapy in the kayakers from the Polish National Team between 22 July and 9 August, 2013.

Day of week	Time of exercise	Type of exercise	Duration (min)	Intensity range*
Monday	10.00 AM	Strength training + specialized on-water training	140	III/V
	16.00 PM	Specialized on-water training	90	II

Tuesday	10.00 AM	Specialized on-water training + stretching	100	I/III
	16.00 PM	Specialized on-water training + 6 km running	110	II/III

Wednesday	10.00 AM	Specialized on-water training + stretching	110	I/III
	16.00 PM	Specialized on-water training + strength training	100	V/II

Thursday	10.00 AM	Ergometer + specialized on-water training + 6 km running	130	II/III
	16.00 PM	Recovery	—	—

Friday	10.00 AM	Strength training + specialized on-water training	120	V/III
	16.00 PM	Specialized on-water training + stretching	80	I/III

Saturday	10.00 AM	Ergometer + specialized on-water training	110	II/III
	16.00 PM	Specialized on-water training + 6 km running	90	II/III

Sunday	10.00 AM	Specialized on-water training	110	III
	16.00 PM	Recovery		

*Lactate acid concentration in blood: I < 2 mmol/L, II < 4 mmol/L, III = 4 mmol/L (lactate threshold), IV > 4 < 6 mmol/L, and V > 6 < 8 mmol/L.

**Table 2:** Oxidative stress indicators and the concentration of urea, cortisol, and testosterone in the blood serum of kayakers from the Polish National Team during a 19-day physical activity including WBC sessions twice a day for the first 10 days.

	Baseline (Day 0)	Day 5	Day 11	Day 19
MDA [10^−2^ nmol/mL]	26.3 ± 2.5	27.7 ± 2.4	26.0 ± 1.8	25.5 ± 1.8
CD [10^−1^ Abs./mL]	4.6 ± 2.5	4.6 ± 1.2	4.4 ± 1.0	3.6 ± 0.8
TBARS [10^−2^ nmol MDA/mL]	50.3 ± 5.0	51.2 ± 7.0	45.9 ± 5.3	42.3 ± 4.4^aabb^
Protein carbonyls [*µ*mol/mg]	538.4 ± 23.3	534.8 ± 136.1	535.4 ± 36.2	535.8 ± 25.5
Vit. E [*μ*g/L]	17.7 ± 8.2	14.6 ± 3.7	13.6 ± 3.3	13.1 ± 2.9
GPx [U/L]	225.9 ± 26.4	191.6 ± 27.8^a^	212.9 ± 42.6	229.4 ± 30.3^b^
TAC [*µ*M CRE]	813.6 ± 67.7	769.6 ± 78.4	774.1 ± 102.0	845.3 ± 114.0
Urea [mg/dL]	33.1 ± 6.3	39.9 ± 6.3^a^	45.2 ± 5.5^aa^	37.2 ± 8.4^c^
Cortisol [nmol/L]	628.2 ± 62.9	661.3 ± 83.1	688.7 ± 73.4	638.4 ± 109.2
Testosterone [nmol/L]	23.8 ± 5.9	22.8 ± 4.9	24.4 ± 4.7	24.9 ± 5.6

The results are expressed as the mean ± standard deviation; ^a^statistically significant difference versus baseline (^a^ *P* < 0.05, ^aa^ *P* < 0.01), ^b^statistically significant versus Day 5th (^b^ *P* < 0.05, ^bb^ *P* < 0.01), and ^c^statistically significant versus Day 11 (*P* < 0.05).

**Table 3:** Selected morphological parameters of the blood of kayakers during a 19-day training cycle with two WBC sessions per day for the first 10 days, one before an exercise bout (morning stimulation) and one after an exercise bout (afternoon recovery). The results are expressed as the mean ± standard deviation. No statistically significant differences were observed.

Morphological blood variable	Term of the study			
	Baseline (Day 0)	Day 5	Day 11	Day 19
RBC (mln/mm^3^)	5.0 ± 0.3	5.2 ± 0.2	5.1 ± 0.2	5.1 ± 0.3
WBC (thous./mm^3^)	5.5 ± 1.1	5.9 ± 1.0	5.6 ± 0.8	5.5 ± 1.1
NEUT (%)	41.6 ± 5.0	44.6 ± 8.1	43.1 ± 4.9	42.4 ± 6.9
LYMPH (%)	44.2 ± 5.0	42.4 ± 7.6	42.7 ± 5.1	44.0 ± 5.9
HGB (g/dL)	15.3 ± 0.7	15.9 ± 0.6	15.4 ± 0.5	15.5 ± 0.8

WBC: white blood cells, RBC: red blood cells, NEUT: neutrophils, LYMF: lymphocytes, and HGB: haemoglobin.
